# Assessment of Nutritional Status and Risk of Malnutrition Using Adapted Standard Tools in Patients with Mental Illness and in Need of Intensive Psychiatric Treatment

**DOI:** 10.3390/ijerph20010109

**Published:** 2022-12-22

**Authors:** Ladina Risch, Florian Hotzy, Stefan Vetter, Sascha Hiller, Kathrin Wallimann, Erich Seifritz, Sonja Mötteli

**Affiliations:** 1 Faculty of Medicine, University of Zurich, 8032 Zurich, Switzerland; 2 Department of Psychiatry, Psychotherapy and Psychosomatics, Psychiatric Hospital of the University of Zurich, 8032 Zurich, Switzerland; 3 Directorate of Nursing, Therapies and Social Work, Psychiatric Hospital of the University of Zurich, 8032 Zurich, Switzerland

**Keywords:** nutritional risk screening, severe mental illness, psychiatric treatment, BMI, nutritional status, malnutrition, depression, schizophrenia, nutrition, diet

## Abstract

People with severe mental illness (SMI) are often in poor physical health, resulting in higher mortality and reduced life expectancy compared to the general population. Although eating habits are one of the main predictors of physical health, few studies assess the nutritional status and eating behavior of people with SMI. The aim of this study was to examine the nutritional status and risk of malnutrition in people with SMI who were in need of intensive psychiatric treatment. The cross-sectional study included 65 inpatients and 67 outpatients with psychotic or depressive disorders from the Psychiatric Hospital of the University of Zurich. Patients’ assessments at admission included anthropometric measurements, such as weight and height, and interview data including severity of symptoms and functioning (SCL-K-9, PHQ-D, CGI, m-GAF), personal and medical data, nutrition risk screening tools (adapted NRS, MNA-SF), and laboratory values. The results showed that 32% of the inpatients and 34% of the outpatients were at risk of malnutrition, which was associated with higher levels of psychiatric symptoms and lower levels of functioning. Regardless, the body mass index (BMI) was overweight in both groups (mean BMI_inpatients_ = 25.3, mean BMI_outpatients_ = 27.9). These results indicate that a substantial proportion of psychiatric patients seems to be at risk of malnutrition, despite most being overweight, and hence they might benefit from nutritional support during their psychiatric treatment. Moreover, nutritional risk screening tools specifically developed for the mental healthcare setting are needed.

## 1. Introduction

Psychiatric disorders, such as schizophrenia, major depression, and bipolar disorders, can lead to serious psycho-functional impairments over time. Moreover, persons with severe mental illness (SMI) often suffer from poor physical health and reduced life expectancy compared to the general population [1,2]. Poor physical health condition is the result of adverse drug reactions of psychiatric medications, as well as chronic diseases, less access to healthcare and unhealthy lifestyles, including alcohol and tobacco abuse, less physical activity, and poor dietary habits [3].

Previous studies have shown that people with SMI have on average a higher caloric intake and a lower diet quality (e.g., higher intake of red and processed meats and refined grains and sweets, along with a lower intake of whole grains, vegetables, and fruits) compared to the general population [4,5,6]. It is assumed that nutritional interventions during psychiatric treatment can improve both physical and mental health conditions [7].

People with SMI face various barriers to obtaining good nutrition, such as medication effects on appetite, reduced motivation levels, sedentariness or agitation, social isolation, cognitive impairments, and financial restrictions [6]. Most of these barriers lead to weight gain [8]. As a result, the risk of cardiovascular diseases, diabetes, and obesity is up to two times higher for people with SMI compared to the general population [7]. Consequently, mental healthcare workers may overlook aspects of malnutrition in their psychiatric patients because such aspects are often masked by patients’ overweight status [9]. Malnutrition is commonly described as a state of imbalanced nutrition, varying from overnutrition to undernutrition due to a diet that is inappropriate for the individuals’ needs. This may lead to changed or decreased body functions [10].

In contrast to psychiatry, the prevalence (and risk) of malnutrition in somatic hospitals has been well researched. In the latter, it is estimated that up to 30% of patients at the time of hospital admission are at risk or suffer from malnutrition [11]. In the hospital setting, malnutrition is mostly considered equivalent to undernutrition [12]. Much evidence shows that nutritional status is important during illness recovery, resulting in shortened hospital stays, lower mortality and readmission rates, and reduced healthcare costs [13,14,15]. As a result, the routine assessment of malnutrition at admission to hospital has been established in many countries [9].

Nutrition risk screening tools should consist of objective and subjective measurements such as physical examination including anthropometric measurements, functional evaluation, dietary habits, and medical history [16]. In particular, to take a preventive approach and to identify patients at risk of malnutrition, it is important to assess the risk factors (e.g., poor appetite, poor mobility and physical activity, substance abuse, and insufficient intake of important food groups including vegetables, fruits, meat, and dairy products) as well as the presence of malnutrition [7,9].

In the mental healthcare setting, however, there exists no specific nutritional risk screening tool [8] and nutritional support or lifestyle interventions are not always available [7,17]. Indeed, the nutritional status and needs of people with SMI have received little attention to date. Among the few studies available, to the best of our knowledge, only one study from the UK explicitly examined the nutritional risk of malnutrition in psychiatric patients [8,18]. It found that 48% of the acute psychiatric inpatients were at risk of malnutrition based on an adapted risk screening tool from general hospital use. In addition, nurses’ judgments about the patients´ nutritional status were not significantly related to the nutritional risk scores [18]. Considering that people with SMI experience various nutritional problems [6,7], but low nutritional support [17], it seems obvious that a substantial proportion of psychiatric patients might be at risk of malnutrition [7,9,18] and that specific nutritional risk screening tools for the mental healthcare setting are required [8].

The aim of this study was to examine the nutritional status of psychiatric patients with SMI using different objective and subjective measures and to estimate the proportion of patients at risk of malnutrition. In addition, we compared the outcomes between inpatients and outpatients who clearly differ, generally, regarding their severity of symptoms. For this purpose, we applied the Nutritional Risk Screening (NRS) [19] and Mini Nutritional Assessment—Short Form (MNA-SF) [20]. These tools have been evaluated and described as two of the major nutrition risk screening and assessment tools for adults in the acute hospital setting [11]. In addition, the MNA-SF also includes important subjective risk factors of malnutrition, as mentioned above [9]. The NRS and MNA-SF were slightly adapted for use in mental healthcare settings and compared with other measures related to nutritional status.

## 2. Materials and Methods

This study was based on cross-sectional interview data and anthropometric measurements related to the assessment of the nutritional status and risk of malnutrition in people with SMI and the need for psychiatric treatment. The full prospective observational study was described in a previously published study protocol [21]. In total, 132 psychiatric patients, including 65 inpatients (Sample 1) and 67 outpatients (Sample 2), were interviewed at the beginning of their treatment at the Psychiatric Hospital of the University of Zurich (Psychiatrische Universitaetsklinik Zuerich (PUK)) in Switzerland.

### 2.1. Participants and Procedure

We included patients aged 18–65 years who were residents in or around Zurich, had psychotic or depressive disorders (classified as F2 or F3 according to the ICD-10 diagnostic tool), and who needed intensive inpatient or outpatient treatment at PUK. Previous research [22] showed that this patient population complies with the criteria for SMI regarding disability and duration of illness [23]. Participants needed sufficient communication skills in the German language and the ability to give written informed consent to participate in the interview. Individuals with a diagnosed eating disorder, patients with imprisonment status, or tourists from foreign countries were excluded.

**Figure 1:**
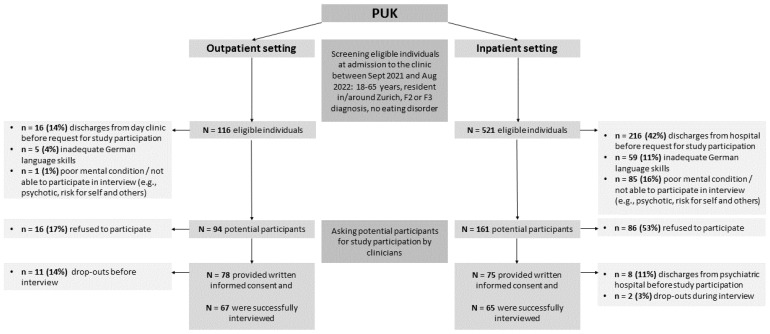
Flowchart of the participants’ inclusion process.

Between September 2021 and August 2022, clinical staff screened eligible individuals regarding gender, age, and main psychiatric diagnosis based on a daily list of all new patients from seven wards for the acute treatment of mental disorders and from a one-day clinic at the PUK. Patients meeting the inclusion criteria were then asked to participate in the study shortly after admission. Upon receiving their agreement and written informed consent, the clinical staff handed over the participants’ contact details to the study team. To obtain balanced samples, we used stratified sampling based on gender and age. Specifically, we included 16 women aged ≤40 years, 16 women >40 years, 16 men ≤40 years, and 16 men >40 years old (for details, see the study protocol [21]), which resulted in a final sample of 65 inpatients and 67 outpatients. The recruitment process is shown in Figure 1.

The participants were invited to study interviews at the PUK, guided by research assistants who had received specific interview training regarding the standardized assessment of anthropometric measures to ensure correct data assessment and recording. Interviews were conducted on average 14 days after the participants’ admissions. The whole interview, as described in the study protocol [21], lasted for an average of 1 h, while this study included only some of the questions. The participants were offered a drink, and they were allowed to take breaks according to their preferences and mental conditions. We did not provide financial compensation. The interview data and anthropometric measures were implemented in the LimeSurvey online survey tool [24]. Using case identification codes, no identifying data were entered into LimeSurvey. The password-protected personal data were only accessible to the core research team.

### 2.2. Measures

As recommended by Reber et al., 2019, different objective and subjective measurements were assessed (e.g., BMI, weight changes, medical history, medication, appetite) along with the calculation of nutritional risk scores to evaluate the patients’ nutritional status.

#### 2.2.1. Anthropometric Measures and Weight Changes

BMI: The BMI was calculated as body weight (kg) divided by height squared (m^2^). Weight (kg) and height (m) were measured to the nearest 0.1 units using the digital scale SOEHNLE 63850 PWD Style Sense Compact 100. Each measurement was repeated twice, and the mean value was calculated. Participants were allowed to wear light clothing, but no shoes.

Waist:hip ratio (WHR): The WHR was calculated as abdominal girth/hip girth. The abdominal girth (m) and hip girth (m) were measured all to the nearest 0.1 units using SECA measuring tape. Each measurement was repeated twice, and the mean value was calculated. Participants were allowed to wear light clothing.

Weight change: The participants were asked about weight loss or gain within the last 3–6 months and (if applicable) the amount (in kg).

#### 2.2.2. Nutrition Risk Screening

Nutritional Risk Screening (NRS): The NRS is a validated and well-established risk screening tool used to identify patients suffering from malnutrition or patients at risk of malnutrition in the hospital setting [16,19]. The nutritional status was assessed by weight loss/BMI/food intake during the last week (0–3 points) and the severity of disease using categories of physical impairment (0–3 points). For the latter, as an adaptation for use in a mental healthcare setting, we used the modified Global Assessment of Functioning Scale (m-GAF), which is a validated tool (ranging from 1 to 100) with good interrater reliability to assess the patients’ mental states [25]. A higher m-GAF score indicates better functioning. The m-GAF scores were assessed using the following categories (0–3 points): no impairment = 91–100, mild impairment = 61–90, moderate impairment = 41–60, and pervasive impairment = 01–40. A total score of 3 points or more in the NRS indicates nutritional deficiencies and a need for nutritional therapy.

Mini Nutritional Assessment-Short Form (MNA-SF): The MNA-SF is also a validated and widely used nutritional risk screening tool in hospital settings and care homes [20,26]. It comprises questions about weight loss, BMI, mobility, psychological stress, neuropsychological disorders (depression and dementia), and reduced food intake due to loss of appetite, or problems with swallowing, chewing, or digestion. In the results, 0–7 points means malnutrition, 8–11 points means to be at risk of malnutrition, and 12–14 points indicates normal nutritional status. Two items were slightly adapted for use in a mental healthcare setting as follows: a) a question about mobility not only bound to physical ability; and b) a question about neuropsychological disorders: severe dementia or depression = 0, dementia = 1, other psychological problems = 2.

Subjective evaluation of nutritional status: The participants were asked to rate their nutritional status (supply of energy and nutrients) on a scale ranging from 1 = very poor to 10 = very good.

#### 2.2.3. Mental Condition

To assess the participants’ mental conditions, we used two subjective and two objective measures.

9-item Symptom-Checklist (SCL-K-9): This checklist is a reliable, efficient, and validated short form of the Symptom-Checklist SCL-90-Revised to assess the participants’ psychopathologic symptomatology and their mental and physical health problems from the last 7 days on a 5-point Likert scale [27]. Higher mean values indicate more serious symptoms. Cronbach’s alpha for the scale in this study was 0.82 (*n* = 132).

9-item Patient Health Questionnaire (PHQ-9): This is a validated and reliable screening tool for depression severity in the last 2 weeks for medical settings [28,29]. The PHQ-9 is the depression element of the PHQ for common mental disorders. The scale ranges from 0 = not at all to 3 = nearly every day. A summed score of 10 points or higher indicates depression (10–14 = slight depression, 15–19 = medium depression, 20–27 = severe depression). Cronbach’s alpha for the scale in this study was 0.79 (*n* = 132).

Global Assessment of Functioning Scale (m-GAF): As described in Section 2.2.2, the participants’ mental states were rated by the interviewers using the following categories (0–3 points): no impairment = 91–100, mild impairment = 61–90, moderate impairment = 41–60, and pervasive impairment = 01–40, while a higher m-GAF score indicated better functioning [25]. In addition to the interview data, we included the m-GAF scores at admission and discharge for the inpatient sample, which were routinely assessed by clinicians at the hospital.

Clinical Global Impression Scale (CGI): The CGI is a brief and validated instrument consisting of two global measures to assess the severity of illness (1) and the degree of improvement since the initiation of treatment (2) [30]. We included only the first measure, which was routinely assessed on a 7-point Likert-type scale ranging from 1 (not ill at all) to 7 (extremely ill) by clinicians at the patients’ admissions.

#### 2.2.4. Biochemical Parameters

We used the following routinely collected laboratory results for the inpatient sample if they were assessed within 8 days after the patients’ admissions: cholesterol, triglyceride, C-reactive protein (CRP), folate acid, and vitamin B_12_. The values were dichotomized into 1 = within reference values, 0 = outside reference values (cholesterol: <5.2 mmol/L, triglyceride: <1.7 mmol/L, CRP: <3 mg/L, folate acid: >7 nmol/L, vitamin B_12_: 150–700 pmol/L). The reference values of the associated laboratory were used to dichotomize the values. No routinely collected laboratory results were available from the outpatient sample.

#### 2.2.5. Personal and Medical Data

The participants responded to additional questions regarding their health, nutrition and sociodemographic variables. In addition, the questionnaire data were supplemented with medical data and selected routine data.

Sociodemographic data: The participants were asked to state their gender, age, education level, housing situation, and source of income. Swiss citizenship was derived from routine medical data.

Medical data: We used the primary psychiatric diagnosis according to ICD-10 (F2 or F3) at the patients’ admissions to the hospital (see inclusion/exclusion criteria). In addition, the participants were asked at what age their first psychiatric problems occurred. The participants’ prescribed medications were derived from the clinical information system. Further, the participants were asked whether they took the medication as prescribed and whether they took additional medications. Based on the participants’ answers, the list of prescribed medications was modified to enable the assessment of the actual intake of medication. According to classification models described in the literature on psychiatric medication [31,32], the prescribed substance classes were assigned to a scale (ranging from 0 = no risk for weight gain to 3 = strong risk for weight gain) and summed up. Consequently, the relevance of potential weight gain could be compared on the scale level. Additionally, we asked the participants whether they took additional nutritional supplements. For the inpatient sample, we also assessed the (in)voluntariness of the admissions based on routine medical data.

Nutrition-related variables: We asked the participants about the presence of nutrition-related diseases (e.g., diabetes, celiac disease, lactose intolerance, food allergies or intolerances, gout, further gastrointestinal diseases), which were diagnosed by a doctor or specialist, as well as other gastrointestinal problems (e.g., swallowing, nausea, diarrhea) and food intolerances that were self-perceived.

The participants were asked to give answers to the following questions on a scale from 1 to 10: Do you believe that nutritional support should be included in the standard psychiatric treatment (1 = not at all, 10 = absolutely)? Has the relationship between mental health and nutrition been given sufficient attention in your previous therapy (1 = not at all, 10 = sufficient)? Answers with 6 points or more were coded as agreements with the question contents. Further, participants were asked whether they had ever had nutrition counselling before or wished to have it now (yes/no). For those who stated ‘yes’, the study team informed the responsible clinicians who then initiated nutrition counselling.

#### 2.2.6. Statistical Analyses

The interview and routine medical data were merged and analyzed in SPSS statistics (version 28) software using case identification codes and data. For the comparison of the samples, a sample size of *n* = 64 patients was calculated for each group (for details, see the corresponding study protocol [21]). First, variables such as BMI, WHR, sum score of prescribed medications and risk scores, as well as SCL-K-9 and PHQ-D scale values, were calculated. The internal consistency reliability of each scale was determined using Cronbach’s alpha. Statistical differences between inpatients and outpatients were determined using *t*-tests for independent samples for continuous data and using chi-square tests or the Fisher–Freeman–Halton exact test for categorical data. If normal distribution was not given, the results were also calculated using the Mann–Whitney U test. In cases of similar results and sufficient cases, the results of *t*-tests were reported. Associations were determined using Pearson correlations. In addition, to determine whether severity of symptoms and weight loss (no = 0, yes = 1) during the last weeks/months could predict the risk of malnutrition, a logistic regression was conducted including control variables such as gender (male = 0, female = 1), age, education (lower educated = 0, higher educated (Matura or higher) = 1), diagnosis (F2 = 0, F3 = 1), and BMI (all variables were entered in a single step). For all analyses, the significance level was set to 5%.

## 3. Results

In total, 521 eligible inpatients and 116 outpatients were screened for study participation (see Figure 1). In both settings, more patients with affective disorders (F3) than psychotic disorders (F2) participated in the study interviews. Nevertheless, refusal to participate was not significantly related to diagnosis, gender, or age (*p* > 0.05). In the inpatient setting, significantly more patients with F2 diagnoses could not participate in the study because of poor mental conditions (*p* < 0.05) and, in the outpatient setting, the new admissions included more F3 diagnoses.

### 3.1. Characteristics of the Participants

Table 1 shows the sociodemographic characteristics of the study participants. The mean ages of the inpatients and outpatients were 39.9 years (SD = 12.9) and 40.2 years (SD = 11.4), respectively. In the inpatient setting, more individuals had only compulsory schooling and were dependent on a disability pension and housing support compared to outpatients. Inpatients also had been affected by mental disorders for a longer period, indicating a higher degree of disability in this group of patients (see Table 2).

Table 2 shows the assessed variables related to the participants’ mental condition and nutritional status. Two-thirds of the inpatients had an F3 diagnosis, compared to 87% of the outpatients. Most of the inpatients and all of the outpatients were voluntarily admitted for psychiatric treatment.

According to the CGI, clinicians rated 68% of inpatients at their admissions as seriously ill and 32% as moderately to significantly ill. In contrast, only 2% of the outpatients were rated as seriously ill at their hospital admissions. At the time of the interview (on average 2 weeks after admission), there were no longer significant differences in the level of functioning (GAF categories) and symptom severity (SCL-K-9) between the inpatient and outpatient groups (see Table 2). However, the PHQ-D scores were significantly higher among the outpatients because of the higher proportion of F3 diagnoses. As per the definition, PHQ-D scores measured only depressive symptoms [28] and, therefore, PHQ-D scores were higher in patients with F3 diagnoses (M = 15.5, SD = 5.5) compared to patients with F2 diagnoses (M = 13.0, SD = 6.6, t = 2.2 [130], *p* = 0.030).

Inpatients took more prescribed medications than outpatients (see Table 2), while participants with F2 diagnoses (*n* = 34) took more prescribed medication than patients with F3 diagnoses (M = 3.4, SD = 1.8 versus M = 2.7, SD = 1.8, t = 1.99 [130], *p* = 0.049). In addition, half of the participants (45% of inpatients and 55% of outpatients) took nutritional supplements, such as vitamin D, vitamin B_12_, magnesium, iron, or a multivitamin preparation, in the last 3–6 months.

### 3.2. Nutritional Status and Risk of Malnutrition

In the inpatient setting, women averaged 67.9 kg (SD = 14.9) and 1.65 m (SD = 0.07), and men averaged 80.6 kg (SD = 18.1) and 1.77 m (SD = 0.07) in weight and height, respectively. In the outpatient setting, women averaged 71.1 kg (SD = 15.0) and 1.62 m (SD = 0.09), and men averaged 92.3 kg (SD = 18.4) and 1.79 m (SD = 0.06). Most participants reported experiencing weight changes during the last 3–6 months (see Table 2). In the inpatient setting, 43% of the patients lost weight before admission (range 1–20 kg), and 28% gained weight (range 1–31 kg). Conversely, 30% of the outpatients lost weight before admission (range 2–40 kg), and 58% gained weight (range 1–20 kg). The BMI was significantly higher in outpatients (M = 27.9, SD = 5.3) than in inpatients (M = 25.3, SD = 5.0), whereas the WHR (M = 0.87, SD = 0.09 for women (*n* = 66) and M = 0.94 m, SD = 0.08 for men (*n* = 66)) did not differ between the settings (see Table 2).

BMI, WHR, and weight change were not related to the type of diagnosis (F2 or F3). For all participants (*n* = 132), BMI was significantly correlated with WHR (r = 0.45, *p* < 0.001), weight gain (r = 0.35, *p* < 0.001), and weight loss (r = −0.27, *p* = 0.002). In addition, for the inpatient setting, a higher BMI was associated with worse laboratory values with proportions above the reference values for cholesterol (r = 0.38, *p* = 0.003), triglycerides (r = 0.39, *p* = 0.002), and CRP (r = 0.40, *p* = 0.001).

In addition to weight changes, a substantial proportion of the participants reported being affected by nutrition-related diseases (e.g., diabetes, celiac disease, food allergies, etc.), gastrointestinal problems, and subjective experienced food intolerances with a higher proportion in the outpatient sample (see Table 2).

The results of the nutrition risk screening tools revealed that a substantial part of the participants were at risk of malnutrition. Based on the adapted NRS, 35% of the inpatients and 37% of the outpatients scored 3 points or higher and, therefore, were at risk of malnutrition. For the adapted MNA-SF, 35% of the inpatients and 28% of the outpatients fell into the category “malnutrition” (0–7 points), 54% of the inpatients and 63% of the outpatients fell into the category “risk of malnutrition” (8–11 points), and 11% of the inpatients and 9% of the outpatients fell into the category “normal nutritional status” (12–14 points). 

Adapted NRS scores were significantly related to adapted MNA-SF scores (Fisher’s exact test = 24.44, *p* < 0.001, *n* = 132). To estimate the overall risk of malnutrition, the scores were combined to make a new risk score: risk of malnutrition was only established if the patients were categorized as “malnutrition” or “risk of malnutrition” in both screening tools. As a result, 32% of the inpatients and 34% of the outpatients were at risk of malnutrition (see Table 2).

Results of the logistic regression showed that the model was significant (χ^2^ = 35.80, df = 7, *p* < 0.001) and a higher risk of malnutrition, as categorized by the new risk score, could be predicted by more severe symptoms (SCL-K-9) and a higher weight loss. Sociodemographic variables (gender, age, education), diagnosis (F2 or F3), and BMI were not significant predictors in the model (see Table 3).

For the inpatients only, we compared the GAF scores assessed by clinicians at admission and discharge (*n* = 14–17 missing values in GAF scores). Inpatients at risk of malnutrition (*n* = 17) had significantly lower GAF scores at admission compared to the *n* = 34 inpatients with no risk of malnutrition (M = 25.7, SD = 8.9 versus M = 34.8, SD = 11.4, t [49] = 2.88, *p* = 0.006). Similarly, inpatients at risk of malnutrition (*n* = 16) had significantly lower GAF scores at discharge compared to the *n* = 32 inpatients with no risk of malnutrition (M = 53.5, SD = 14.9 vs. M = 63.4, SD = 11.8, t [46] = 2.51, *p* = 0.015). Treatment duration was on average 35 days, with no differences related to the risk of malnutrition.

From a subjective perspective, outpatients rated their nutritional status as significantly lower than inpatients (see Table 2). Interestingly, the subjective evaluation of nutritional status was not related to the objectively assessed risk score (*p* > 0.05). In addition, 39% of the inpatients and 51% of the outpatients wished to receive nutrition counselling, while half of the patients already had previous experiences with nutrition counselling. Moreover, 82% of the inpatients and 87% of the outpatients agreed that nutritional support should be included in the standard psychiatric treatment. However, 69% of the inpatients and 71% of the outpatients stated that the topic had not been given sufficient attention in their treatment to date.

## 4. Discussion

To the best of our knowledge, this is the first study to comprehensively assess nutritional status and the risk of malnutrition in a sample of inpatients and outpatients with severe mental illness. Based on an adapted risk score from the somatic hospital setting, our results showed that a third of psychiatric patients were at risk of malnutrition. Being at risk of malnutrition was associated with a higher severity of symptoms and lower functioning. A substantial proportion of the patients were affected by various nutritional problems, such as weight gain, weight loss, dyslipidemia, diabetes, gastrointestinal problems, and food intolerances.

The impact of nutritional status on recovery from illness, mortality, and treatment complications such as the length of hospital stays and the rate of readmissions has long been recognized [11,13,14,15]. Routine assessment of malnutrition at the patient’s admission to a somatic hospital is widely used, but it is still missed in other settings, such as outpatients or between the change of settings [9]. In psychiatry, due to the predominance of obesity- and cardiovascular-related nutritional problems [4,5,6,7,8], the risk of malnutrition has been completely neglected to date [8,18]. In addition, no specific nutritional risk assessment tool exists for the psychiatric setting [8].

Therefore, in this study, we used a combination of two established and validated nutritional risk scores of the somatic hospital setting (NRS and MNA-SF), which we slightly adapted in order to estimate the risk of malnutrition in psychiatric patients. The results showed that 32% of the inpatients and 34% of the outpatients exhibited a risk of malnutrition. These results are in line with the average prevalence rate of 30% in somatic settings using the same risk scores [11], but were lower than those in the study by Abayomi and Hackett (2004), who indicated a risk of malnutrition in 48% of psychiatric patients using a different nutritional risk score [18].

Our results showed no differences in the risk of malnutrition between inpatients and outpatients at the time of the interviews, which took place about 14 days after the patients’ admissions. At the time of the interviews, the severity of symptoms (SCL-K-9) and levels of functioning (GAF categories) were comparable in both samples. However, the results could be different if the risk of malnutrition was directly assessed at the patients’ admissions with usually worse levels of symptoms and functioning for inpatients (as indicated by CGI in Table 2).

The improvement in the inpatients’ symptoms between their admissions and interviews is consistent with the clinical effect of the intensive inpatient treatment. In addition, the risk of malnutrition was not related to sociodemographic variables or diagnoses (F2 or F3). These associations have to be further investigated with samples of more diverse patients, for instance, by including elderly patients and those with other diagnoses, such as addiction. However, the results showed that the risk of malnutrition was associated with lower levels of functioning (GAF) in inpatients at admission and discharge from the hospital. In line with previous research from the hospital setting [13,14,15], these findings show that nutritional status has an important impact on recovery from mental illness. Therefore, during hospital treatment, adequate nutritional support might be a cost-effective approach for successful recovery. Overall, according to the patients’ views, nutritional support should be included in the standard psychiatric treatment, but it has not been sufficiently addressed so far. This mismatch between desire and availability has already been described in previous research [7,17].

Furthermore, the results of the logistic regression analysis showed that the risk of malnutrition could be predicted by weight loss and the severity of symptoms. Both predictor variables were assessed by independent measures (self-reported weight loss, SCL-K-9) from those used to assess the risk of malnutrition (BMI, intake, GAF).

On the one hand, these results validated the applied nutritional risk screening tools and showed that they are an easy and efficient way to detect patients at risk of malnutrition. On the other hand, and even more importantly, these results also indicated that BMI was not a significant predictor of the risk of malnutrition. As most psychiatric patients are affected by obesity- and cardiovascular-related nutritional problems [4,5,6,7,8], the risk of malnutrition might be overlooked. This may be the reason why the subjective evaluation of nutritional status was not related to the objectively assessed nutritional risk score, similar to the results of the study by Abayomi and Hackett (2004) [18].

In general, people with SMI face various nutritional barriers [6] often associated with weight gain [8]. In line with these findings, our study revealed that 80% of the participants reported weight changes during the last 3–6 months. Outpatients, in particular, seemed to be affected by problems of weight gain, and their BMI (M = 27.9) was significantly higher compared to inpatients (M = 25.3), although the latter group had a higher risk for medication-induced weight gain based on the calculated sum score.

However, due to the cross-sectional study design, the effect of medication on weight could not be assessed. For the inpatient sample, a higher BMI was associated with laboratory values above the reference values for cholesterol, triglyceride and CRP, all known cardiovascular risk factors [33,34]. In summary, the majority of psychiatric patients seemed to be affected by nutritional problems leading to weight gain or weight loss. Therefore, future studies are needed to develop a comprehensive nutritional risk screening tool for the specific requirements of the mental healthcare setting [35]. In addition, further longitudinal studies are needed to further examine the relationship between the severity of symptoms and risk of malnutrition in psychiatric patients.

A limitation of our study is that we included more patients with depressive (F3) than psychotic (F2) disorders because there were more F3 admissions in the outpatient sample, and in the inpatient setting, more patients with F2 diagnoses were not able to participate due to a poor mental condition. For this patient group, the structure and/or duration of the whole interview (for more details, see study protocol [21]) might have been inappropriate. Although we did not find relevant differences between F2 and F3 diagnoses in relation to our research question, the imbalance in diagnosis might reduce the generalization of the results.

Another limitation is that the study is partly based on self-reports, and these may not be accurate. In addition, in the inpatient setting, there might have been more participants with a genuine interest in the topic, in contrast to the outpatient sample, which had a very high response rate. Further, we had no routinely collected laboratory values in the outpatient setting, and the correlation between laboratory values and BMI could have only been shown in the inpatient sample.

## 5. Conclusions

The results of this study showed that most patients with SMI are affected by nutritional problems, while inpatients and outpatients seem to have different nutritional needs. On average, inpatients are more affected by weight loss, and outpatients are more affected by weight gain. Despite the predominance of overweight and associated risk factors, there was a substantial proportion of participants at risk of malnutrition: 32% of inpatients and 34% of outpatients, as predicted by the severity of symptoms and weight loss.

Consequently, we recommend the development of a specific risk screening tool for the mental healthcare setting, which includes items covering overnutrition and undernutrition (e.g., gain or loss of appetite, weight gain or loss) and other important factors such as medication and severity of symptoms. Routine assessment of patients’ nutritional status should be established as part of psychiatric treatment in the same way it is done in the somatic hospital setting, using validated tools rather than subjective evaluations conducted by clinical staff or the patients themselves.

The last and most important point is that psychiatric patients should receive adequate nutritional support during their psychiatric treatment, and its access/availability in mental healthcare treatment should be improved.

## Figures and Tables

**Table 1:** Characteristics of the participants.

Sociodemographic Variables	Inpatients (*n* = 65)		Outpatients (*n* = 67)	
	*n*	%	*n*	%
Gender, female	32	49	34	51
Age > 40 years	32	49	34	51
Swiss nationality	46	79	45	79
Education				
Compulsory schooling	14	22	9	13
Vocational education	22	34	30	45
Matura (high school exit exam)	7	11	6	9
Higher vocational education	8	12	8	12
University	14	21	14	21
Source of income				
Salary	21	32	32	47
Disability pension	18	28	5	8
Social-welfare benefits	12	18	15	22
Support by family	9	14	10	15
Savings	3	5	5	8
Unknown	2	3	0	0
Housing situation				
Alone	21	32	29	43
Together with others	37	57	38	57
Residential care home	6	9	0	0
Homeless	1	2	0	0

Note. Swiss nationality: *n* = 7 missing values for inpatients, *n* = 10 missing values for outpatients.

**Table 2:** Variables related to the participants’ mental condition and nutritional status.

Variables	Inpatients (*n* = 65)		Outpatients (*n* = 67)			
	*n* or M	% or SD	*n* or M	% or SD	Chi-Square or *t*-Test	*p*
Main psychiatric diagnosis (F3)	40	62	58	87	10.81	0.001
Age of first occurrence of psychiatric problems (years)	22.7	12.3	28.0	15.3	2.17	0.032
Involuntary admission (IA)	14	23	0	0		
CGI at admission to hospital					65.81	<0.001
Moderately ill (4)	4	7	9	15		
Significantly ill (5)	15	25	49	83		
(Extremely) seriously ill (6–7)	40	68	1	2		
GAF categories (interviews, in average 14 days after admission)					3.64	0.162
Mild impairment (GAF 61–90)	17	26	12	18		
Moderate impairment (GAF 41–60)	31	48	43	64		
Pervasive impairment (GAF 01–40)	17	26	12	18		
SCL-K-9 (higher scores = more psychiatric symptoms)	1.8	0.8	2.0	0.9	1.31	0.192
PHQ-D (higher scores = more depressive symptoms)	13.4	6.3	16.3	5.1	2.83	0.005
Prescribed medication						
Number of medications	3.7	2.0	2.1	1.2	5.39	<0.001
Sum score (higher scores = negative effect on weight gain)	2.9	2.7	1.6	2.0	3.35	<0.001
BMI (kg/m^2^)	25.3	5.0	27.9	5.3	2.92	0.004
WHR (m)	0.9	0.1	0.9	0.1	1.02	0.310
Weight gain in last 3–6 months						
Proportion (%)	18	28	39	58	12.52	<0.001
kg	1.9	4.8	3.7	4.7	2.16	0.033
Weight loss in last 3–6 months						
Proportion (%)	28	43	20	30	2.49	0.114
kg	2.6	4.4	3.0	6.6	0.43	0.672
Risk of malnutrition (combined adapted NRS and MNA-SF)	21	32	23	34	0.06	0.806
Subjective evaluation of nutritional status (1–10 = better)	6.6	2.3	5.5	2.2	2.97	0.004
Selected routine laboratory results (proportion within reference values)						
Cholesterol (<5.2 mmol/L)	35	58	na	na		
Triglyceride (<1.7 mmol/L)	41	70	na	na		
C-reactive protein (<3 mg/L)	41	67	na	na		
Folate acid (>7 nmol/L)	52	93	na	na		
Vitamin B_12_ (150–700 pmol/L)	51	90	na	na		
Nutrition-related diseases (e.g., diabetes, celiac disease, food allergies)	12	19	24	36	5.01	0.025
Gastrointestinal problems (e.g., swallowing, nausea, diarrhea)	16	25	8	12	3.56	0.059
Food intolerances	21	32	34	51	4.62	0.032

Note. *n* = frequency, M = mean, Na = data not available; in case of *n* < 5 per cell, the Fisher–Freeman–Halton exact test was used instead of the chi-square test; IA: *n* = 5 missing values; CGI: *n* = 6 missing values in inpatients and *n* = 8 missing values in outpatients; routine laboratory results: missing values between 6% and 14%.

**Table 3:** Predictors of the risk of malnutrition.

Risk of Malnutrition			
Variables	B	SD	OR (95% CI)
Constant	−2.21	1.60	0.11
Gender	0.24	0.44	1.27 (0.53–3.02)
Age	0.03	0.02	1.03 (0.99–1.07)
Education	−0.48	0.47	0.62 (0.25–1.55)
Diagnoses (F2 or F3)	0.13	0.51	1.14 (0.42–3.09)
BMI	−0.07	0.05	0.94 (0.86–1.03)
Weight loss	1.91	0.46	6.75 (2.76–16.52) **
SCL-K-9	0.65	0.27	1.91 (1.13–3.21) *
−2LL	132.24		
Omnibus test	χ^2^ = 35.80, df = 7, *p* < 0.001		
Nagelkerkers R^2^	33%		
Classification accuracy	74%		

Notes. *n* = 132, B = unstandardized regression coefficient, SD = standard deviation, OR = odds ratio, −2LL = −2 log likelihood; * *p* < 0.5, ** *p* < 0.001.

## Data Availability

The dataset generated for the current study is available from the last author (S.M.) upon written request.
